# Exploring Trade-Offs for Online Mental Health Matching: Agent-Based Modeling Study

**DOI:** 10.2196/58241

**Published:** 2024-10-01

**Authors:** Yuhan Liu, Anna Fang, Glen Moriarty, Cristopher Firman, Robert E Kraut, Haiyi Zhu

**Affiliations:** 1 Department of Computer Science Princeton University Princeton, NJ United States; 2 Human-Computer Interaction Institute Carnegie Mellon University Pittsburgh, PA United States; 3 7 Cups Wilmington, DE United States

**Keywords:** agent-based modeling, mental health, algorithmic matching, social computing, online communities

## Abstract

**Background:**

Online mental health communities (OMHCs) are an effective and accessible channel to give and receive social support for individuals with mental and emotional issues. However, a key challenge on these platforms is finding suitable partners to interact with given that mechanisms to match users are currently underdeveloped or highly naive.

**Objective:**

In this study, we collaborated with one of the world’s largest OMHCs; our contribution is to show the application of agent-based modeling for the design of online community matching algorithms. We developed an agent-based simulation framework and showcased how it can uncover trade-offs in different matching algorithms between people seeking support and volunteer counselors.

**Methods:**

We used a comprehensive data set spanning January 2020 to April 2022 to create a simulation framework based on agent-based modeling that replicates the current matching mechanisms of our research site. After validating the accuracy of this simulated replication, we used this simulation framework as a “sandbox” to test different matching algorithms based on the deferred acceptance algorithm. We compared trade-offs among these different matching algorithms based on various metrics of interest, such as chat ratings and matching success rates.

**Results:**

Our study suggests that various tensions emerge through different algorithmic choices for these communities. For example, our simulation uncovered that increased waiting time for support seekers was an inherent consequence on these sites when intelligent matching was used to find more suitable matches. Our simulation also verified some intuitive effects, such as that the greatest number of support seeker–counselor matches occurred using a “first come, first served” protocol, whereas relatively fewer matches occurred using a “last come, first served” protocol. We also discuss practical findings regarding matching for vulnerable versus overall populations. Results by demographic group revealed disparities—underaged and gender minority groups had lower average chat ratings and higher blocking rates on the site when compared to their majority counterparts, indicating the potential benefits of algorithmically matching them. We found that some protocols, such as a “filter”-based approach that matched vulnerable support seekers only with a counselor of their same demographic, led to improvements for these groups but resulted in lower satisfaction (–12%) among the overall population. However, this trade-off between minority and majority groups was not observed when using “topic” as a matching criterion. Topic-based matching actually outperformed the filter-based protocol among underaged people and led to significant improvements over the status quo among all minority and majority groups—specifically, a 6% average chat rating improvement and a decrease in blocking incidents from 5.86% to 4.26%.

**Conclusions:**

Agent-based modeling can reveal significant design considerations in the OMHC context, including trade-offs in various outcome metrics and the potential benefits of algorithmic matching for marginalized communities.

## Introduction

### Background

People are increasingly turning to online mental health communities (OMHCs) for mental and emotional support [1,2]. OMHCs are a practical and accessible way for users to receive both informational and emotional support on a variety of mental and emotional concerns [3], with communities offering general support for any need or support for specified health issues. For example, communities such as 7 Cups provide general 1-on-1 peer counseling chats, whereas platforms such as BabyCenter provide targeted support resources for pregnant women [4,5]. OMHCs have been found to be vital in maintaining and improving people’s well-being, such as reducing depression, fostering meaningful relationships, and increasing trust in mental health treatment.

However, despite the ability of OMHCs to yield meaningful and positive relationships between users, they currently rely on naive methods (ie, “first come, first served” and solely based on topic of discussion) for members to find these relationships without consideration of users’ unique characteristics and preferences. Prior work suggests that current matching systems do not adequately support users’ needs and capabilities; ineffective matching can also lead to fewer long-term relationships and reduced member commitment [4]. Moreover, this lack of purposeful matching methods may be particularly harmful for marginalized communities, who have both strong preferences for mental health care providers with a similar background and particular reliance on online communities for support [6-10]**.** Given these challenges, intelligent forms of matching can provide more optimal matches with minimal effort on a user’s end [4].

However, matching is a complicated mechanism design problem [11,12]. It is challenging to meet all the possible matching goals between support seekers and providers, and prioritizing one goal might lead to worse outcomes in other goals. Given these challenges, tools such as agent-based simulation that have long been used to apply social science theories to the design of human-computer interaction systems are useful for revealing the complexities and various trade-offs in matching protocols for online community designers [13]. Importantly, running these low-cost, virtual experiments can predict community members’ likely reaction to alternative design choices without disrupting existing community dynamics. Thus, agent-based modeling enables researchers and community designers to pin down factors leading to desirable outcomes and understand how design choices affect behavioral outcomes by modeling the intervening processes [14].

### Goal of This Study

In this study, we answered the following research question: How can we experiment with new matching algorithms for OMHCs? Specifically, we sought to experiment with these new algorithms without harming or disrupting the existing community. To do this, we created a simulated “sandbox” based on agent-based modeling that allows community stakeholders to play with different matching algorithms and helps designers consider complex trade-offs in building new mechanisms for their community. To build this simulation based on a real OMHC, we collaborated with one of the world’s largest peer support platforms. We used the platform’s data set to accurately replicate the platform in our simulation and then experiment with alternative protocols in the simulation to analyze how these new matching policies affect users’ experiences. We created and tested seven new algorithmic matching policies based on prior work studying OMHC users’ needs: (1) *first come, first served;* (2) *last come, first served*; (3) *similarity-based* matching, which uses cosine similarity to prioritize support seekers and support providers of similar features; (4) *gender-based matching*; (5) *age-based matching*; (6) *topic-based* *matching*; and, finally, (7) *filter-based* matching, which focuses on protecting teenagers and gender minority groups.

The contribution of our work is to show the application and example use of agent-based simulation to uncover the effects of alternative policies in the design of online community matching. Exploring different matching protocols has the potential to disrupt the particularly sensitive population of OMHC users through implementation and iteration processes; given this, our work showcases the benefits of instead using agent-based simulation to reveal the impacts of various algorithms. In addition to applying simulation to the OMHC context, we contribute practical findings for matching design in OMHCs, such as how optimizing based on topic can improve chat experiences for vulnerable communities, as well as trade-offs, such as how using algorithmic matching can increase the quality of conversations but also the waiting time for support seekers.

### Relevant Prior Work

#### Online Mental Health Support

Social support through online platforms has been shown to improve users’ well-being in numerous ways, such as reducing depression, lowering suicidal ideation, spreading information about mental health, and enabling help seeking for stigmatized populations [3,15,16]. Most online mental health support takes place in OMHCs, where peers can speak anonymously about their experiences for free and 24/7, which is essential for groups who particularly struggle with stigma and access to resources [17], such as adolescents and lesbian, gay, bisexual, transgender, and queer (LGBTQ+) populations [18-22]. However, some groups, such as female individuals and gender minority groups, also face unique challenges of sexual and verbal harassment while chatting on OMHC platforms [4]. Thus, special consideration for protection of vulnerable groups is crucial in building better matching mechanisms given the unique benefits and challenges they face. Our study shows how agent-based simulation can help community designers build and test ways for people, including minors and gender minority groups who have specific needs, to find relevant and useful partnerships when engaging in online mental health support.

#### Matching for Mental Health Purposes

As people perceive those similar to themselves to be more trustworthy and likely to share their worldviews, research has thoroughly supported that a client and therapists’ race, language, gender, and other variables impact therapeutic outcomes [23-26]. In traditional mental health resources, clients have strong preferences for choosing a therapist of the same race [27] and their same gender [23]. Effects of racial matching may be especially important for minority groups, such as Black clients, given mitigation of general mistrust toward mental health services [28,29]. Topic and content have been found to be important in care for client satisfaction and therapy quality [30-33]. In terms of the online context, our work builds on previous literature by Fang and Zhu [4] that found that gender, age, and experience level are all significant factors in people’s preferences for online support relationships; in particular, gender minority groups being matched with those of similar gender identity and avoiding support providers who were significantly younger resulted in support seekers having a more positive experience. In fact, users consistently share their gender and age with one another when trying to find online support relationships, thus further showing the need for and lack of more efficient forms for matching. We note that, apart from the work by Fang and Zhu [4], most of the prior work has studied important matching features in the traditional therapy context. Our work builds on this previous knowledge by showcasing evaluation on community-level outcomes when matching based on important features (age, gender, and topic) in the *online* context specifically.

#### Agent-Based Modeling and Online Community Design

Agent-based modeling is the simulation of the actions of agents to understand their behaviors and interactions under different conditions, has been useful for informing the design of online communities through simulating how different design choices impact desired outcomes, and has been used in past work to explore topics such as social influence and information propagation [13,34,35]. One other primary benefit of agent-based modeling, as opposed to direct experimentation, is that effects can be observed over long periods as one can run the agent-based model repeatedly and for lengthy time cycles; thus, downstream and even unintended effects (rather than just first-order effects) can be identified [14]. Importantly, agent-based modeling serves as a testing ground for community designers and researchers to surface how different theories affect the community broadly but also allow them to isolate the factors leading to particular outcomes [14]. For example, Ren and Kraut [13,36] have shown how agent-based modeling can be used to apply social science theories and understand trade-offs in design decisions; they applied these methods to explore motivations for online community participation and how different moderation methods affect discussion in online communities. Given the proven power of agent-based modeling for understanding online community dynamics, we applied agent-based modeling to the online mental health context to showcase its usefulness for exploring matching algorithms and understanding trade-offs in these design decisions.

## Methods

### Research Site

To study algorithmic matching and simulation in the real online mental health context, we collaborated with one of the largest existing support platforms that is currently an active and growing community. This online platform provides free 24/7 chat support and has >54 million members and 500,000 trained volunteer counselors. Users who sign up to seek support—whom we will call “support seekers”—can chat in 1-on-1 chat rooms with trained “volunteer counselors.” Volunteer counselors complete a roughly 1-hour, psychology-based training that is based on active listening and motivational interviewing skills. Counselors can also receive awards on their profiles from completing additional, optional training modules, such as specialized courses for specific conditions (eg, attention-deficit/hyperactivity disorder and depression) as well as advanced general skill courses (eg, “Active Listening” and “Managing Emotions”). Note that, although volunteer counselors have some training on the site, we refer to our study’s platform as a peer support platform given that all counselors are nonprofessional and only lightly trained and members can be both support seekers and counselors.

All users are required to provide their age to the platform, whereas other demographic information (eg, gender) is optional. The primary support method on the research site is through 1-on-1 chats between one support seeker and one volunteer counselor. The current matching process is a self-selection by volunteer counselors in which support seekers send a request to join a live queue and wait to be picked by a volunteer counselor to begin a chat. Support seekers also have the option to select among “topic tags” (eg, “depression” or “relationship stress”) but are not required to do so. No other information about support seekers besides their waiting time and possibly their topic tag is displayed in the queue. We will later use features such as people’s demographic information and topic choices in our study’s matching protocols. Support seekers can cancel their chat request at any point and may do so especially if they are waiting too long.

### Data

The data set consists of all chat messages between January 2020 and April 2022, which includes 8 million chats with >1.5 million support seekers and >288,000 volunteer counselors. All chat data include the anonymized message text, time stamp, and user IDs involved. The data set also includes users’ sign-up dates and birth years. Note that no personally identifiable information about users is available. Relevant to our study, chats can also be rated by support seekers from 1 to 5 stars once the chat has continued for a certain length of time or after the chat ends; volunteer counselors are not able to rate a chat. In addition, users can block one another at any point, including during their conversation.

### Ethical Considerations

This study used behavioral log data obtained through a collaboration with the studied OMHC to conduct our analysis, and data collection followed Health Insurance Portability and Accountability Act (HIPAA) and confidentiality agreements. All users who register on the site are informed of and accept the use of their anonymized data for research. All data were anonymized before analysis, and no personally identifiable information was used in this study. Note that chat messages were only analyzed to find the gender distribution of users to generate agents for simulation purposes, which is described in the Building Agent-Based Simulation: Assumptions section. One author of this paper worked at a 3-month internship for this study’s research site. This work has been exempted from ethical approval as our study does not constitute human subject research according to the Carnegie Mellon University institutional review board (STUDY2019_00000488), and no users of the OMHC were directly interacted with for this study; no additional consent or compensation to users was needed for this research.

### Summary of Methods

**Figure 1:**
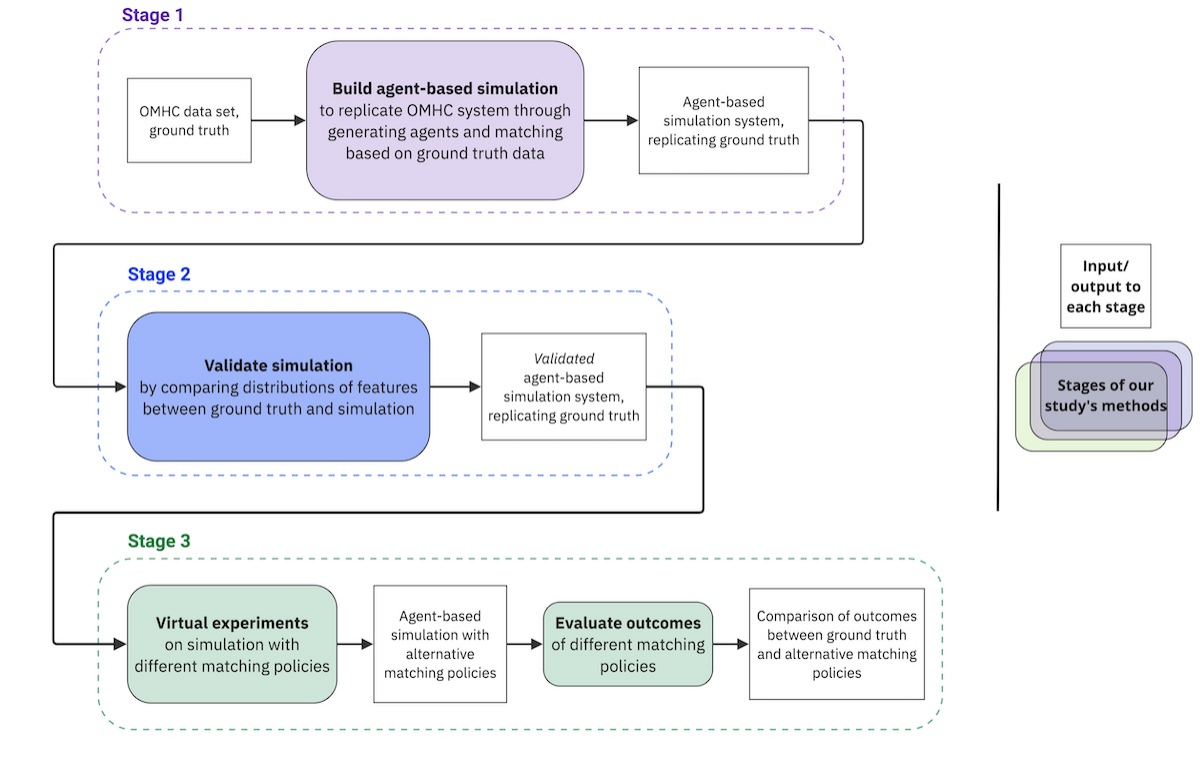
Workflow diagram showing inputs and outputs for the three stages of building and experimenting with agent-based simulation: (1) building an agent-based simulation, (2) validating the agent-based simulation, and (3) conducting virtual experiments using the validated simulation. OMHC: online mental health community.

To showcase how agent-based modeling can be used to experiment with matching policies in the online mental health context, we followed 3 stages, as outlined in Figure 1.

### Building an Agent-Based Simulation: Assumptions

#### Overview

We first built a simulation based on agent-based modeling to replicate the current matching mechanisms of our study’s research site and used outcome prediction models to validate this replication. In the following sections, we outline the assumptions made to build our simulation, all of which were implemented based on the real OMHC data set. Overall, we found that there was an extremely high correlation (ie, Pearson coefficient) between our simulation and the real research site. As all features had a high correlation, in the following sections, we show figures for only a few features as visuals of how our simulation performed compared to the research site.

#### Simulation Period

In our agent-based simulation, both support seekers and volunteer counselors have the possibility of being matched during each “round” or simulation period. We determined that a simulation period of 1 minute was fit for our model as 1 minute is temporally granular enough to yield quick matching of users and derivable from our empirical data.

#### Generation of Agents

**Figure 2:**
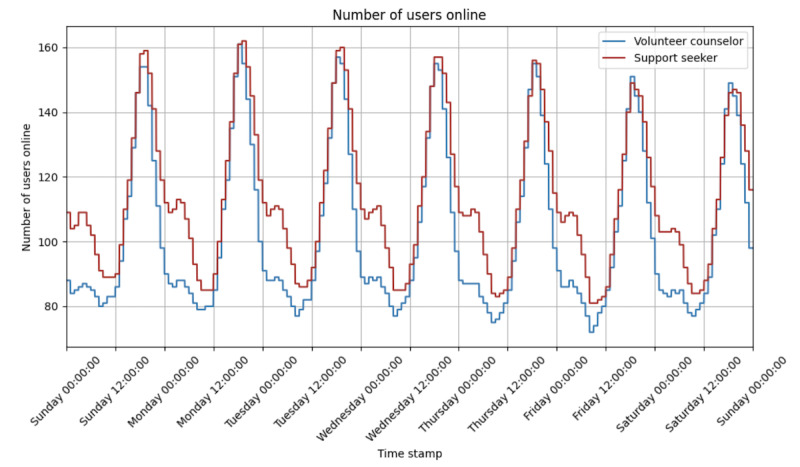
A line graph showing the number of support seekers and volunteer counselors who are online in each simulation period. The number of support seekers always exceeds the number of volunteer counselors, with the number of support seekers and volunteer counselors who are online at any given minute ranging between 81 and 162 (mean 113.26, SD 22.56) and between 72 and 161 (mean 102.49, SD 25.07), respectively.

Our simulation consisted of 2 types of agents: support seekers and volunteer counselors. Each simulation period (ie, each minute) generates new support seeker and volunteer counselor agents that are eligible for matching. Agents are considered “online” (ie, available to chat) immediately when they are generated. To determine the number of agents generated, we analyzed our study’s data set from January 2020 to April 2022 to find the average number of online support seekers and volunteer counselors for each simulation period over a week (ie, 10,080 min; Figure 2).

**Figure 3:**
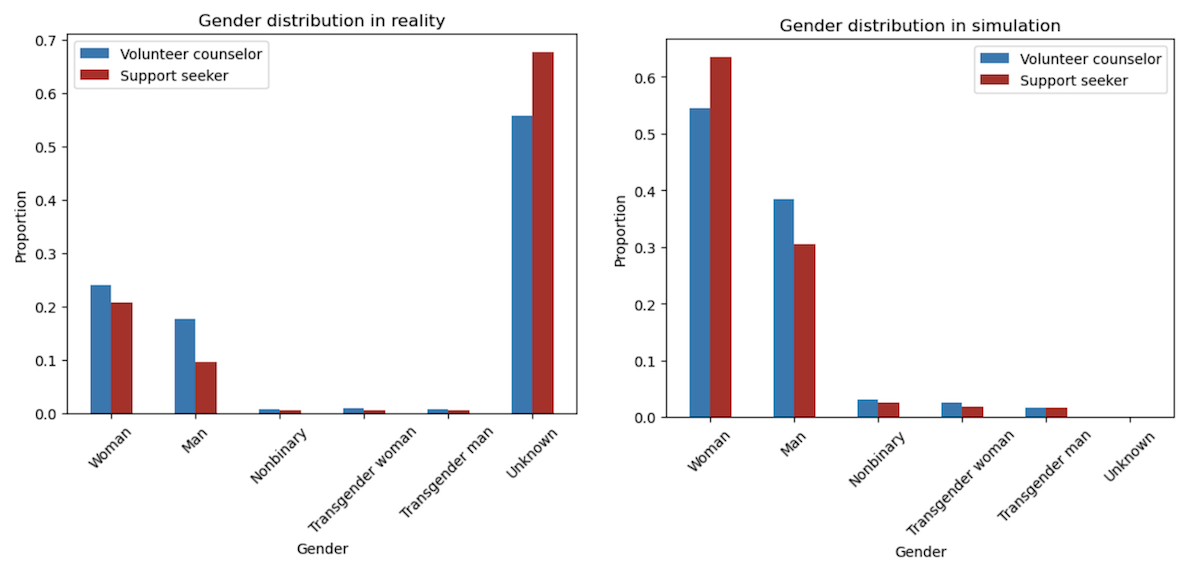
Sex distribution in reality (left) versus our simulation (right). In the ground truth data set, many support seekers and counselors had unknown gender. In contrast, all agents were assigned a gender in our simulation. We assigned gender according to the gender distribution of known genders (female, male, nonbinary, transgender female, and transgender male) in the ground truth data set.

When generated, each agent is also given several personal characteristics that may be significant to matching (gender, birth year, and topic of interest) [4]. We analyzed the OMHC data set to find the distribution of these characteristics at each simulation period and assign agents’ characteristics so that the simulation’s distribution was identical to the distribution found in the real OMHC data. Birth year was part of the raw data set, giving us complete and accurate real data to draw from. However, we had to conduct a labeling process for users’ gender identity as a minority of users input their gender on their profile manually. Following prior work, we labeled gender according to whether a user had self-identified their gender in chat logs (ie, “I am a female” or “I am non-binary”), which has been found to be highly accurate and only mislabel gender for 0.8% of OMHC users [4]. Using this process, we were able to label the gender of 35% of support seekers and 50% of volunteer counselors in our data set. We then applied the distribution of gender among those whose gender was known to our generated agents so that all agents had a gender characteristic. This distribution is shown in Figure 3. In terms of topic, we used a previously built and validated topic classifier [37] on all chats in our data set to find the distribution of topics. The topic classifier we used from the work by Wang et al [37] was built using the same data set as our study. Using Empath [38], a tool that uses neural word embeddings for generating and validating lexical categories in large-scale text data, Wang et al [37] tuned Empath’s model by feeding in “seed words” of 18 popular topics that support seekers discuss. The top 18 topics are *romantic relationships*, *dating*, *pandemic*, *self-improvement*, *suicide*, *depression*, *parents*, *anxiety*, *family*, *stress*, *lonely*, *overwhelming*, *sexuality*, *LGBTQ*, *intimacy*, *home*, *dissociative identity*, and *health*. Similar to assignment of the previous personal characteristics, we applied the real OMHC data set’s distribution of topics to assign support seeker agents a topic of interest and counselor agents a list of up to 3 topics of interest that they have more experience or interest in talking about. The most frequent topic discussed on the site in our data set was “self-improvement,” followed by “dating,” “parents,” and “depression.” Our simulation’s frequency distribution among all topics was the same, with a Pearson correlation coefficient of 1.

#### Patience Level

As support seekers may leave the site while waiting for a chat, we also replicated the “patience level” of support seekers. All support seeker agents were given a number of minutes that they are willing to wait to be matched before they cancel their request (ie, leave the platform). Support seeker agents go offline when the number of simulation steps in which the support seeker remains unmatched exceeds their patience level. Similar to previous characteristics, we assigned patience levels to support seeker agents according to the distribution we found from analyzing the data set for how long support seekers wait until canceling their chat requests in the queue. We assigned support seeker agents a patience level according to the distribution of time for support seekers to cancel their chat requests in the data set. The mean patience level in reality is 4.15 (SD 3.26) minutes, whereas our simulation’s patience level had a mean of 4.16 (SD 3.27) minutes. The Pearson correlation coefficient was 0.991.

#### Chat Length

Once a support seeker and volunteer counselor are matched, their chat length is set in minutes, and volunteer counselor agents go offline after a chat ends. We set the chat length in our simulation to follow the distribution of conversation length found in the log data. The distribution of chat length in reality found through log data versus our simulation’s distribution had a Pearson correlation coefficient of 1. The mean chat length in reality is 17.67 (SD 15.44) minutes, whereas our simulation’s chat length had a mean of 17.67 (SD 15.42) minutes.

#### Matching Support Seekers and Volunteer Counselors

Finally, we describe the decision of matching a support seeker and volunteer counselor together to chat.

**Figure 4:**
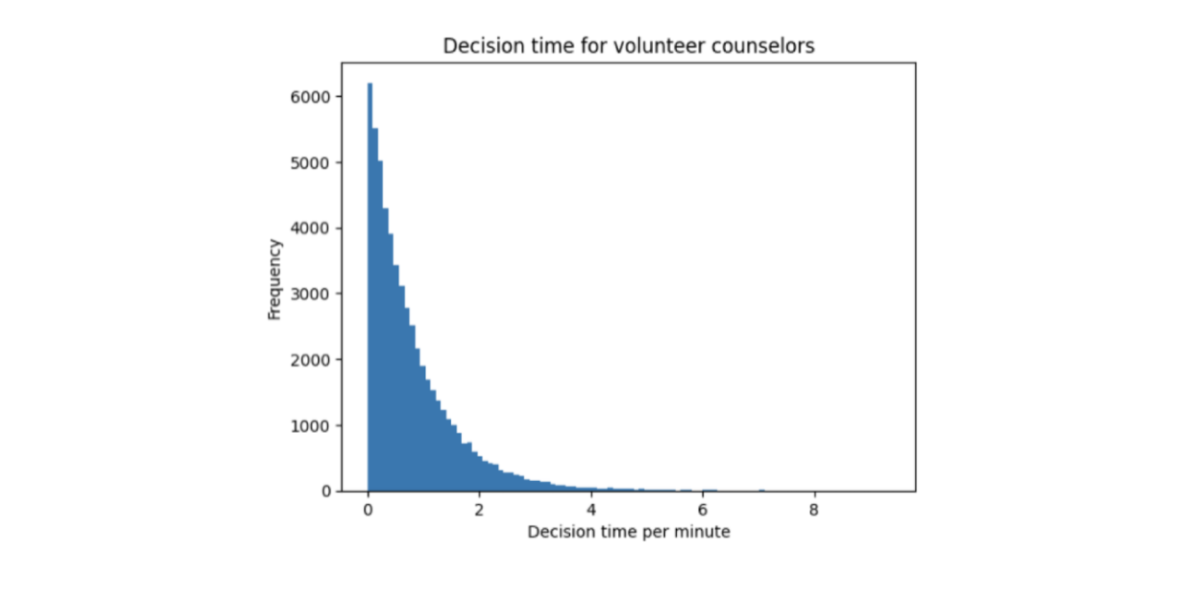
Distribution of decision time of volunteer counselors, which is modeled using an exponential distribution with λ of 1.25.

All agents who are online and not chatting in the current simulation period are considered available to be matched in the current round. In each simulation period, all volunteer counselor agents are presented with a list of all available support seeker agents and pick a support seeker to chat with depending on the matching policy. In the replication of the research site’s system, volunteer counselors pick a support seeker to chat with randomly following an exponential distribution model for the time it takes to make their choice (Figure 4).

### Building an Agent-Based Simulation: Prediction Models

#### Overview

Although there are several outcome metrics that could be used to assess a support seeker’s experience in a chat, the ability of a support seeker to give the chat a rating of 1 to 5 stars is the most direct indicator of the match. In addition, past work has found that users place more value on avoiding the worst chat experiences rather than aiming for the best given that extremely negative chats may include bullying and harassment and also may deter users from returning to the site [4]. Blocking another user is the main action that users take when they have a bad chat on the research site and is the most obvious reflection of a negative support seeker and volunteer counselor relationship. Both support seekers and volunteer counselors on our study’s OMHC can block each other through the chat interface and provide a reason for blocking.

Note that, although our data set includes good indicators of matching quality, there are no existing data that can be used to calculate matching quality for simulated results. Therefore, we created 2 outcome prediction models—a chat rating prediction model and a blocking prediction model. In the validation process, we used those 2 models to test whether our replicated simulation was an accurate representation of the current system. In our virtual experiments, we used these prediction models to evaluate the effectiveness of our designed matching algorithms as well.

#### Chat Rating Prediction

We gathered all chat ratings in the data set, with 80% of samples from the data set used as our training set whereas the remaining 20% were used as our testing set. We balanced the training set using the synthetic minority oversampling technique, which generates artificial samples for minority classes based on existing samples using the k-nearest neighbor algorithm [39]. After using the synthetic minority oversampling technique, we ended up with 16,730 samples for each of the 5 chat rating classes (1 to 5 stars).

As independent variables in our chat rating prediction, we used inputs of both volunteer counselors’ and support seekers’ gender, birth year, and topic. Our experiments included random forest, logistic regression, support vector machine, and decision tree models to find the best model to predict chat rating. Note that accuracy in this context means that the output must equal the true chat rating and is considered incorrect if it outputs any of the other 4 chat ratings. Given that random forest outperformed all other models in accuracy and *F*_1_-score at a 0.72 accuracy and 0.72 *F*_1_-score, we used the random forest classifier for our chat rating prediction model.

#### Blocking Prediction

In creating our training data set for building the blocking prediction model, we labeled a support seeker and volunteer counselor pair as 1 if at least one person blocked the other and as 0 otherwise. Similar to our chat rating prediction model, we used input fields of gender, birth year, and topic. For all agents, we used an 80%/20% split of samples from the data set for training and testing, respectively. We proceeded again with random forest classification as it resulted in the best performance, with the highest precision and recall scores of 0.91 accuracy and 0.91 *F*_1_-score.

### Validation of the Agent-Based Simulation

During the validation phase, we calculated and compared distributions of the features in the following sections between the real OMHC system data and our simulation’s data. We then reported the Pearson correlation coefficients [40] between the real and simulated data distributions.

#### Number of Users Online

**Figure 5:**
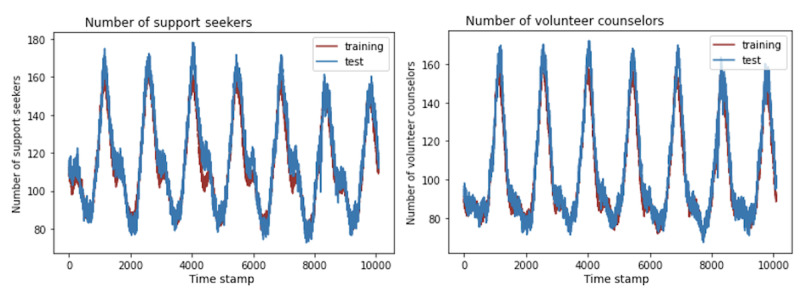
Distribution of number of support seekers (left) and volunteer counselors (right) in the training and test sets. The Pearson correlation coefficient of the number of support seekers between the training and test sets was 0.974, whereas the Pearson correlation coefficient of the number of volunteer counselors between the training and test sets was 0.982.

To validate the replication of the OMHC system, we split the data set into a 6-month training set and a 2-month test set. Figure 5 compares the number of online support seekers and volunteer counselors in each minute between the training set and test set in a period of 1 week. We found similar distributions between the training and test set, with Pearson correlation coefficients of 0.974 and 0.982, respectively.

#### Chat Ratings

**Figure 6:**
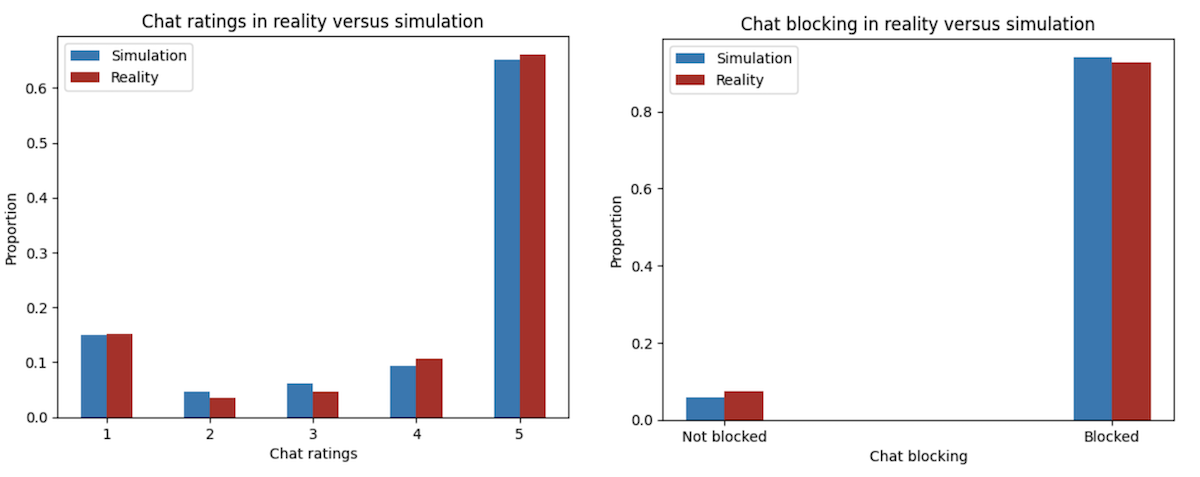
Comparison of chat ratings (left) and blocking (right) between reality and our simulation. Our simulation’s proportions of 1-star to 5-star ratings (shown in blue) were, respectively, 14.96%, 4.64%, 6.08%, 9.26%, and 65.06%, whereas the ground truth’s proportions (shown in red) were 15.18%, 3.51%, 4.56%, 10.63%, and 66.12%, respectively. Regarding pairs that resulted in blocking, the ground truth proportion of blocked support seeker and volunteer counselor pairs was 5.3% compared to our simulation’s proportion of 5.86%.

We used our chat rating prediction model to compare the distribution of chat ratings between our simulation and the ground truth. We found that we accurately simulated rating distributions on the research site, with a Pearson correlation coefficient of 0.99 between our replication and the ground truth (Figure 6). Similarly, we validated our replicated system using the blocking prediction model. Using the blocking prediction model, we found similar distributions of pairs that engage in blocking to those in the ground truth, with a Pearson correlation coefficient of 0.949, as shown in Figure 6, leading to further confidence that our simulation is an accurate representation of the current platform’s system.

#### Waiting Time

In terms of waiting time for support-seekers in our simulation, we found that the distribution of waiting time for support seekers who are matched with a volunteer counselor was similar to the distribution in the real online community system, although with a lower SD. The mean waiting time in reality is 3.2 (SD 2.9) minutes, whereas our simulation’s waiting time had a mean of 3.2 (SD 2.02) minutes. The Pearson correlation coefficient was 0.995.

#### Matching Rate

We also compared the proportion of support seekers who were matched with a volunteer counselor to the total number of support seekers available to be matched. Note that support seekers may cancel their chat request if they are unmatched for longer than their patience level. To evaluate the ground truth, we analyzed the proportion of chats taken by volunteer counselors on the research site to the total number of requests by support seekers in the research site queue. We found that our simulation resulted in an overall 78.35% matching rate in a week, whereas the ground truth showed an average of 83.27% matching rate across all weeks in our data set. Although our simulation’s matching rate was slightly lower than that of the ground truth, the likely explanation is that our simulation framework only allows support seekers and volunteer counselors to engage in one chat at a time, whereas the research site allows volunteer counselors to take multiple chats at once if they desire. Given this, we found that our simulation’s matching rate was acceptable and still closely resembled the actual state of the research site.

### Virtual Experiments

Next, we applied our simulation as a “sandbox” to test different matching algorithms. In the following sections, we review the matching algorithms in our experiment and their outcomes on the metrics of chat ratings, blocking, waiting time, and matching rates.

#### Applicant-Proposing Deferred Acceptance Algorithm

Our research problem consisted of 2 types of agents (support seekers and volunteers) with preferences and personal characteristics for matching. As a result, we considered it akin to the stable marriage problem, which seeks to find a stable matching between 2 classes of elements where both sides have an ordering of preferences. Thus, we used the *applicant-proposing deferred acceptance algorithm* as our matching algorithm [11,41], adapted from the established matching method for New York public schools [42].

In each simulation period, there are 2 sets of agents: support seekers (M={m_1_, m_2_,..., m_n_}) and volunteer counselors (L={l_1_, l_2_,..., l_n_}). Each support seeker has an ordered preference list—P(m)={l_1_, l_2_,..., l_m_}—where a support seeker’s first choice is volunteer counselor l_1_, their second choice is volunteer counselor l_2_, and so on.

1. Each support seeker “applies” to their highest-ranked volunteer counselor according to their preferences, and each volunteer counselor “holds” their highest-ranked application and rejects the rest.
2. At any stage at which a support seeker has been rejected, they “apply” to their next most preferred volunteer counselor whom the support seeker agent prefers (if one remains). Each volunteer counselor holds their most preferred set of applications and “rejects” the rest.
3. The algorithm stops when no rejections are issued and each volunteer counselor is matched to the applicants they are holding.

Any agent who is not matched in this simulation period is marked as “waiting” and continues to be available for matching in the next matching period (along with any newly generated agents). Multimedia Appendix 1 shows the pseudocode for the aforementioned algorithm.

#### Matching Algorithms

##### Overview

The key design choice in algorithmic matching is to construct the preference lists of support seekers and volunteer counselors. Unlike school or physician matching with limited options, hundreds of volunteer counselors are available for any support seeker at any given time. As it is impractical to ask each support seeker to rank each counselor (and vice versa), preferences must be generated using rules or prediction models. The 7 algorithms are described in this section.

We chose methods based on our previous understanding of the research site support seekers’ needs and consultations with the research site’s leadership, including the chief executive officer and lead engineer. In general, the research site team expressed that their priorities were to optimize satisfaction on the platform (ie, chat rating) without reducing the general matching rate. It was important to the research platform that matching protocols allow for better experiences for support seekers but also that the research site could continue to serve most of its large support seeker population. Platform leadership was also particularly interested in gender-based protocols given that gender was a key factor in how people chose volunteer counselors. In addition, a “hard filter”–based protocol was suggested to try to mitigate harassment. Community leaders and our research team decided against directly optimizing for outcome metrics, such as rating-optimized or blocking-minimized protocols, for validity reasons; our study’s prediction models (refer to the Building Agent-Based Simulation: Prediction Models section) use these same metrics (eg, rating and blocking), which would create validity issues if we were to make evaluations on protocols that directly optimize for these metrics.

Each algorithm varies in how it “recommends” a support seeker to each volunteer counselor in each simulation period—volunteer counselors have a 90% chance of taking the recommendation and a 10% chance of random selection.

##### First Come, First Served

Support seekers are ranked in volunteer counselors’ preference lists by decreasing waiting time. The goal of “first come, first served” is to improve the number of successful matches and prioritize serving support seekers who arrive to the queue first [43].

##### Last Come, First Served

Support seekers are ranked in volunteer counselors’ preference lists by increasing waiting time. The goal of “last come, first served” is to minimize queue size when support seekers are more likely to leave the queue the longer they wait [43].

##### Similarity-Based Matching

We used 3 dimensions of access to us that have been shown in previous literature to be important for matching purposes: *gender*, *age*, and *topic* of discussion. Using gender, age, and topic, we defined a vector for each agent with their gender identity, birth year, and topic of interest to calculate 2 agents’ similarity using the cosine similarity between their vectors. Agents with higher similarity are ranked higher in other agents’ preference lists.

As reviewed previously, past work has found that clients often seek therapists similar to themselves among multiple dimensions. In terms of *gender*, prior work has found that it is one of the most important factors in choosing a support provider in online platforms in both client preferences and outcomes, and especially so for gender minority groups [4,44]. In addition, people who are closer in *age* (especially for older populations) are better suited for one another in OMHC support [4] given their similarity in experiences and communication. Finally, having a therapist that is knowledgeable and willing to discuss the client’s needs and issues is vital to the therapeutic relationship. *Topic* relevance is a widespread idea in the space of online communities; for example, social media sites regularly infer a user’s interests to recommend them relevant content (eg, purchase suggestions and personalized advertising) using someone’s previous behaviors on the site [45]. Similarly, in our case, we judge a counselor’s expertise on topics based on their past chats. Each support seeker is assigned a topic of interest that they wish to chat about according to distribution based on the real OMHC data set. On the basis of the top 3 topics by frequency for each volunteer counselor in our OMHC data set, we assign each simulated volunteer counselor a top-3 topic list. Volunteer counselors with a relevant topic in their list are ranked higher in the respective support seekers’ preference list, and vice versa.

In addition, we include matching protocols exploring each of these 3 dimensions (gender, age, and topic) individually.

##### Age-Based Matching

Support seekers and volunteer counselors who are closer in age to each other are ranked higher in each other’s preference lists.

##### Gender-Based Matching

Support seekers and volunteer counselors with the same gender identity are ranked higher in each other’s preference lists.

##### Topic-Based Matching

Volunteer counselors are prioritized in a support seeker’s preference list if the counselor’s topic list contains the support seeker’s topic of interest; similarly, support seekers are prioritized in volunteer counselors’ preference lists if the topic of interest is in the counselor’s expertise. In other words, if a support seeker’s topic is in a counselor’s topic list, then we label a feature of “expertise matching” as 1 (and 0 otherwise) and use expertise matching as the only matching criteria.

##### Filter-Based Matching

Suggested by stakeholders in our study’s research site, we implemented filter-based methods to prioritize the protection of 2 vulnerable groups—namely, in this context, teenagers and gender minority groups. The filter-based method includes 3 different pools for agents: underaged (aged ≤18 years) pool, gender minority (noncisgender women or noncisgender men) pool, and all others. Volunteer counselors can only select support seekers who are in the same pool as themselves. However, apart from this limitation on what support seekers are available to volunteers, volunteer counselors can pick anyone (ie, random selection) following the existing protocol on the site’s simulation (refer to the Matching Support Seekers and Volunteer Counselors section).

## Results

### Overview

Our study’s primary contribution is the application of agent-based simulation to the online mental health context. However, we also review in the following sections the outcomes of experimenting with 7 specific algorithms using our simulation sandbox, shown in Tables 1-3. Full table results can be found in Multimedia Appendix 1. Although these are just 7 possible protocols for matching in this context, in the following sections, we review our comparison of their outcomes to show the kinds of findings and trade-offs revealed through an agent-based simulation.

**Table 1:** Outcome metric results for different algorithms’ performance. Significant outcomes when compared to the replication of the research site (first row) are indicated.

		Rating, mean	Blocked pairs (%)	Matching success rate (%)	Waiting time for matched clients (min), mean	Waiting time for unmatched clients (min), mean
Replication of the research site		4.05	5.86	78.91	3.19	3.69
First come, first served		4.06	5.82	81.93^a^	3.68^a^	2.73^a^
Last come, first served		4.04	6.11	74.81^a^	2.61^a^	4.69^a^
**Similarity based**		4.04	7.37^a^	79.36^b^	3.34^a^	3.53^a^
	Age based	4.02^a^	7.22^a^	79.97^a^	3.40^a^	3.41^a^
	Gender based	4.07^b^	6.04	81.81^a^	3.61^a^	2.86^a^
	Topic based	4.31^a^	4.26^a^	80.70^a^	3.45^a^	3.29^a^
Filter based		4.03^b^	6.21^b^	61.30^a^	3.24^a^	3.86^a^

^a^*P*<.001.

^b^*P*<.05.

**Table 2:** Average chat rating results for different algorithms’ performances among different groups. Listed are the mean ratings for each group. Significant outcomes when compared to the replication of the research site (first row) are indicated. Our simulations showed that the similarity-based protocol (and its subprotocols) and the filter-based protocol had significant effects on the average chat rating among demographic groups. For example, both filter- and similarity-based protocols raised the average chat rating for minority groups but lowered it for nonminority groups.

		Adults	Underaged group	Non–gender minority group	Gender minority group
Replication of the research site		4.00	4.28	4.06	3.80
First come, first served		4.01	4.31	4.07	3.84
Last come, first served		3.98	4.31	4.05	3.83
**Similarity based**		3.95^a^	4.46^a^	4.04^a^	4.20^a^
	Age based	3.94^a^	4.39^a^	4.03^a^	3.76
	Gender based	3.94	4.32	4.06	4.14^a^
	Topic based	4.26^a^	4.53^a^	4.33^a^	4.06^a^
Filter based		3.92^a^	4.39^a^	3.98^a^	4.69^a^

^a^*P*<.001.

The outcome metrics used to evaluate the algorithms are defined as follows:

1. Average rating—average rating for all pairs in the simulation predicted by the rating prediction model (the higher the better)
2. Percentage of blocked pairs—percentage of pairs in the simulation that were predicted as “blocked” (the lower the better)
3. Matching success rate—the ratio of support seekers who were successfully matched (ie, chatted with a volunteer counselor) to all support seekers in the simulation (the higher the better)
4. Average waiting time (matched)—average waiting time of support seekers before they were matched in the simulation (the lower the better)
5. Average waiting time (unmatched)—average waiting time of support seekers whose waiting time exceeded their patience level and quit (the higher the better)

We ran 1-tailed *t* tests to see whether different algorithms’ outcome metrics were statistically significant when compared to the replication protocol. We indicate both the standardized *P* value threshold of .05 as well as a more conservative threshold of .001 in the tables, focusing our discussion on the findings with *P*<.001.

**Table 3:** Blocking results for different algorithms’ performances among different groups. Shown are the percentage of pairs that resulted in blocking behavior from either party. We indicate statistically significant findings. Our simulations showed that the similarity-based protocol helped reduce blocking for underage and gender minority individuals but raised blocking for adults and the non–gender minority group. The topic-based protocol showed a statistically significant reduction in blocking for adults, the underaged group, and the non–gender minority group. The filter-based protocol yielded better results only for the gender minority group, with a worse performance for all other groups.

			Adults (%)		Underaged group (%)		Non–gender minority group (%)		Gender minority group (%)
Replication of the research site			6.73		1.85		5.45		12.31
First come, first served			6.66		1.91		1.86		12.5
Last come, first served			7.03		1.86		5.66		13.13
**Similarity based**			8.69^a^		1.25^a^		7.34^a^		8^a^
	Age based	8.29^a^		1.88^a^		6.77^a^		14.32^b^	
	Gender based	7.02		1.52		5.89^a^		8.64^a^	
	Topic based	4.97^a^		0.99^a^		3.76^a^		11.84	
Filter based			7.38^a^		2.35^a^		6.66^a^		0.44^a^

^a^*P*<.001.

^b^*P*<.05.

### Average Rating

Overall, the results of our virtual experiments followed intuition in that different matching policies served different goals. Average chat rating was one of the metrics of the most interest to our research team in evaluating algorithm outcomes given its interest to our community stakeholders as a good proxy for community satisfaction on the site. We found 2 reliable (*P*<.001) results in which the topic-based protocol yielded significantly higher ratings than the replication protocol and the age-based protocol had significantly lower ratings.

The topic-based protocol performed significantly better than the replication of the research site (*P*<.001) but also all other algorithms. While all other algorithms output average ratings between 4.02 and 4.07 out of 5 stars, the topic-based protocol had a 4.31 average rating out of 5 stars—yielding a statistically significant increase of 6% over an already high baseline replication. Regarding the age-based protocol, it had a statistically significant but marginal decrease in average chat rating of 0.7% compared to the replication. Generally, we found that the age-based protocol performed well for minors (Table 2) but did not have any improvement for adults (who make up most of the population). As a result, age similarity seems to matter for young people connecting but not for the general adult population. We discuss more about the breakdown of protocols across demographic groups in the Results by Demographic section.

### Blocking

In general, blocking rates remained low for all protocols, including in the replication. However, we did observe one statistically significant improvement among protocols, particularly when using the topic-based protocol to match pairs; the proportion of support seeker and volunteer counselor pairs who engaged in blocking (from either party) was reduced from 5.86% to 4.26%. On the other hand, similarity-based matching (combining age, gender, and topic) and the age-based protocol yielded a marginal increase in the blocking rate.

### Matching Success Rate

Regarding overall matching success rate, we found that first come, first served, as expected, led to the highest matching success rate, whereas last come, first served had one of the lowest matching success rates. Given that last come, first served as a matching protocol is aimed at keeping queue size small but not necessarily serving the most people, it follows intuition that we observed a low waiting time for matched pairs but overall relatively fewer successfully matched pairs (we explore this further in the Waiting Times section).

Worth noting is that all protocols other than similarity-based matching showed statistically significant results (*P*<.001) compared to the replication when it came to the matching success rate. First come, first served and age-based, gender-based, and topic-based matching were all protocols that resulted in higher matching success rates. Last come, first served and filter-based mechanisms resulted in statistically significantly lower matching success rates for the community. Filter-based matching resulted in the most striking difference—a 22.3% reduction in overall matching success rate; although we find in Table 2 and discuss later in this paper that there are many benefits to a hard filter–based mechanism for certain groups, our simulation showed a substantial trade-off when it came to the number of people in the community able to find support chats.

### Waiting Times

Waiting times were generally within 1 to 2 seconds of each other when comparing across protocols. However, we note an intriguing finding in that first come, first served initially showed counterintuitively one of the *longest* waiting times for people who were successfully matched. However, upon further reflection, this result actually was to be expected. First come, first served matched the greatest number of people; therefore, more people with higher patience levels and who, thus, had *higher waiting times* were also able to be matched. This increased the average waiting time of successful matches. Thus, the average waiting time for *unmatched* agents only included the agents who were impatient (short patience levels); as a result, the average waiting time for matched clients seemed higher, and the average waiting time for unmatched clients seemed lower. The opposite case occurred for last come, first served; there were short waiting times for successfully matched support seekers, but few support seekers were successfully matched. Thus, we note for community stakeholders that evaluating the average waiting time requires consideration of the matching success rate in conjunction rather than being evaluated in isolation.

### Results by Demographic

We show in Tables 2 and 3 a breakdown of different algorithms’ performances for average chat rating and blocking percentage by demographic groups of adults, underaged people, and non–gender minority groups. Note that we do not have racial information for this analysis, so we are limited to commenting on the effects on only gender and age matching for this study. We also provide the results for matching success rate and waiting times in Multimedia Appendix 1. In general, the largest differences were observed for underaged people and gender minority groups, showing that algorithmic matching has the greatest potential effect for these often marginalized, excluded, and vulnerable groups.

When breaking down protocols by their performances among different groups, we observed several trade-offs on how algorithms perform on majority versus minority groups. It also allowed us to see that the current state of the research site has drastically different effects on various demographic groups. For blocking rate, we noted that the replication of the research site had a significantly higher blocking rate of 12.31% among gender minority groups—an alarming rate that is more than double that of non–gender minority groups. This follows prior work finding that LGBTQ+ communities on OMHCs have a higher risk of harassment [5]. This finding also allowed us to see the major degree of improvement allowed for by the algorithmic matching as we found that the filter-based algorithm (which restricts gender minority support seekers to only being paired with also gender minority counselors) led to a striking improvement of 96% for blocking (from 12.31% to 0.44%) and 23% for average chat rating. Thus, our simulation results give quantitative support to previous qualitative findings that have suggested that LGBTQ+ support seekers prefer a counselor of a similar identity [5]. In particular, the reduction in blocking behavior is promising regarding protecting minority or vulnerable groups from experiencing unwanted behaviors or relationships.

There are significant trade-offs with algorithm choice that emerged from our breakdown of results by demographic group. As reviewed previously, the filter-based algorithm performed exceptionally when it came to gender minority groups. It resulted in an impressive average chat rating of 4.69 out of 5 stars for gender minority groups, a 23.4% improvement over the replication protocol. It also predicted a very low proportion of pairs who blocked one another for both minors and gender minority groups (2.35% and 0.44%, respectively, compared to the replication’s results of 6.73% and 12.31%, respectively). However, it notably performed poorly overall when it came to chat rating and blocking among adults, underaged groups, and gender majority groups. In fact, the filter-based algorithm resulted in a *worse* performance compared to the replication of the research site for all other groups for both chat rating and blocking; this indicates that the hard filter approach results in worse user experiences for most of the site’s population compared to if there was no algorithmic consideration in matching at all. This result may complement previous work that found that LGBTQ+ users in OMHCs feel safer speaking with other LGBTQ+ users [4] but also reveals a likely trade-off for lower overall satisfaction among majority groups on a platform such as the research site. We observed a similar trade-off when it came to the overall similarity-based model. Compared to the replication of the research site, the similarity-based protocol showed a significant decrease in average chat rating for adults and non–gender minority groups (–1.3% and –0.4%, respectively) but a significant increase for underaged people and gender minority groups (+4% and +10.5%, respectively). Regarding blocking, there was an almost equal rise in blocking rates for adults and non–gender minority groups (+29% and +35%, respectively) as there was a decrease in blocking rates (a positive result) for underaged people and gender minority groups (–32% and –35%, respectively). However, note that, except for gender minority groups, blocking rates were relatively low among all other groups to begin with.

However, despite this, one of the most notable takeaways from our simulation results is that this trade-off between the experiences of minority and majority groups was not necessarily the case with all protocols. We found that the topic-based protocol performed well overall even among the marginalized communities we studied despite no consideration of gender or age in its matching criteria and improved the experiences of the general community. When compared to the replication protocol, we found that all results of the topic-based protocol, with the exception the insignificant finding for the blocking rate of gender minority groups, were improvements on *both* chat rating and blocking among *all* groups; other outcomes similarly showed improvement or a similar performance to that of the replication of the research site, as shown in our full results in Multimedia Appendix 1. Regarding underaged people, the topic-based protocol (average chat rating of 4.53 and blocking rate of 0.99%) actually outperformed the hard filter–based protocol (average chat rating of 4.39 and blocking rate of 2.35%), which is the most restrictive protocol for prioritizing the matching of demographic groups. As a result, a key takeaway from these results is that directly using demographics of gender and age as matching criteria is not necessary to improving the experiences of the targeted groups. Although topic-based matching does not include any demographic consideration, it still led to significant improvement for groups that are marginalized or vulnerable due to their demographics (age and gender) and shows that improving the experiences of minority groups is not necessarily exclusive to improvement for the overall population as well. However, we note that our findings of demographics’ effects on matching are limited to gender and age; racial matching is an important factor for traditional services, but our analysis lacked enough racial data, and thus, we cannot draw conclusions on the role of any racially or culturally based matching.

## Discussion

### Agent-Based Modeling for Designing OMHCs

In this work, we showcased our important contribution of applying agent-based simulation to the OMHC context and provided a framework for how it can help the creators and designers of online communities weigh the various trade-offs when building mechanisms to best help their support seekers find meaningful relationships online. Our study suggests that different goals can be achieved through different algorithmic choices for these communities, from optimizing the quality of conversations to the protection of gender and age minority groups on these platforms.

Our research was guided by previous literature that revealed how algorithmic matching is particularly beneficial in the online mental health context and the numerous considerations that are necessary for effective partnerships to aid users’ well-being [4,46]. Through simulating the algorithms and mechanisms of the research site, we found that there are numerous trade-offs to be made in deciding how to match users together in OMHCs. For example, communities aiming to optimize the number of users that engage in chats may wish to experiment with first come, first served methods given simulation results that it has the highest overall matching rate. Alternatively, algorithmic matching may lead to better conversations between users but also increase the time that users must wait for a match. Our simulation revealed that there is indeed substantial improvement that can be made in the quality of conversations using algorithmic matching, such as a >6% increase in the average chat rating just using topic-based matching. However, users may become impatient while waiting for a “best fit” chat partner and log off the site, and thus, fewer people overall are helped by the platform. We see this reflected in our experiments—for example, first come, first served is able to match the most people, with a matching rate of nearly 82% despite being the simplest algorithm without optimization for users’ characteristics.

We also considered and offered the benefits of agent-based modeling in conversations with the stakeholders of the research site. Although these conversations introduced other important considerations in algorithmic matching implementation, such as the computational resources and refactoring of the codebase to implement matching, the stakeholders of our study’s research site found our results insightful and beneficial to the community. In particular, the site’s leadership expressed that simulating algorithmic matching helped them weigh the effects and trade-offs of different design choices for their site without disrupting the existing community through continuous algorithmic testing. Other conversations were also had between our research team and the site’s leadership, such as which outcome measures were most crucial in evaluating the platform’s success for support seekers. The research site has begun developing an algorithmic matching system for their platform based on this work.

It is also worth noting that the research site’s stakeholders wanted to know more about the reasoning behind the numerical results for each matching algorithm. As a result, we believe that it is important that the presentation of these experiments is accompanied by model explanation and algorithmic transparency as well to best help community stakeholders understand results and make decisions based on these types of simulations. Indeed, this suggestion is supported by a plethora of past research that has found that model transparency is necessary for trust and understanding when deploying new algorithms in online communities for both community decision makers and community members [47-49]. We have released our simulation as open source (refer to the Data Availability section).

### Matching Criteria for Online Support Chats

It is important to note that there does not exist a universally best design for all communities; instead, the choice of algorithms and mechanisms for mental health communities is specific to the community’s context, its goals, and its support seekers [37,50]. However, although our experiments are not necessarily generalizable to every OMHC, they do provide some initial insights into making algorithmic choices for these communities.

Past work in traditional mental health services (eg, therapy) has found that using demographics for matching clients and providers is important for outcomes, particularly so for minority and vulnerable groups [23,24,44]. Our findings in the online community context support this notion in that matching based on gender and age indeed improved outcomes for underage and gender minority groups. However, a more surprising result from these experiments in our agent-based simulation model is that matching approaches that do not use demographics as matching criteria can also serve to protect vulnerable groups. As discussed, we found that matching solely based on topic resulted in high average ratings and low blocking percentages among all groups and was an improvement on the baseline. Indeed, topic-based matching outperformed for average rating, proportion of blocking, and the matching success rate when compared to the similarity-based protocol that also included age and gender as matching criteria. However, it is possible that using race or cultural background as a matching criterion could lead to improvements given their importance in research on matching in traditional mental health contexts. However, regarding our findings on gender and age, topic as an effective metric for matching people in vulnerable groups makes some intuitive sense; people who are teenagers, for example, are likely to share similar issues compared to older adults (eg, parenting or divorce), and people who are from gender minority groups may also use an OMHC to seek support for gender issues, transitioning, or other general LGBTQ+ questions [4]. As a result, topic may inherently connect people who not only are of similar demographics but also have similar *experiences* to one another—this includes those who are not necessarily in what our simulation considered a vulnerable group (minors or gender minority groups), and thus, we observed the topic-based protocol performing well on the overall population (Table 1) largely due to its improvement for majority groups in addition to minority groups (Tables 2 and 3). It is important to note the privacy risks when it comes to people’s personal information given to online platforms, and there is some evidence that people have reservations when it comes to revealing this information [4]; thus, our work importantly reveals that bettering people’s experiences is not necessarily reliant on more demographic or other personal information about them.

We note another surprising result that using similarity between people among all features—age, gender, and topic—in our similarity-based protocol generally did not result in significant improvements compared to other protocols. Supporting prior work on the importance of demographic features [4,23,24,44], our evaluation of outcomes by demographic features (Tables 2 and 3) showed improved experiences for the group that a protocol used as a matching feature (ie, the gender-based protocol for gender minority groups or the age-based protocol for minors). However, the *general* similarity-based protocol that used all features (age, gender, and topic) only showed marginal improvement among underaged and gender minority groups when it came to blocking proportions (Table 3), albeit slightly more substantial improvements for the same groups when it came to average chat rating (Table 2). When evaluating the findings in Tables 2 and 3, we hypothesize that simply matching on all characteristics is not necessarily conducive to an overall improvement for vulnerable groups; instead, intentional choice must be made for selecting certain features (such as gender for gender minority groups) to aid their experience. Overall, we found that just as much or even more improvement was observed for vulnerable groups when solely matching on one characteristic, such as gender for gender minority groups or age for underaged people, and even more so that topic was as good or even better as a sole matching criterion compared to cosine similarity among all features. However, we also acknowledge a potential issue in our simulation’s use of cosine similarity as it may not be the best way to measure similarity between people in this context; further work may be necessary to test other similarity measurements. In addition, as mentioned previously, the lack of racial and cultural background data for our study may have limited our findings.

### Limitations

Our work has several limitations. First, although our study’s primary contribution is using agent-based modeling to show how we can simulate algorithmic outcomes for OMHCs, we note that we cannot replicate the complex systems of these communities completely. Second, chat ratings and blocking are just 2 possible outcome metrics to evaluate the performance of a support seeker and volunteer counselor pair. These measurements have limitations; for example, volunteer counselors are unable to rate chats, and support seekers may use the 1-to-5–star scale differently. There are other possible metrics that may have been good measurements of the performance of a support seeker and volunteer counselor pair. For example, the research site periodically sends out emotional wellness tests to support seekers to evaluate how their mental health has improved over time; unfortunately, during our data collection period, support seekers rarely completed these tests, and thus, we did not have adequate data to analyze these results for our simulation. Retention is another metric that could be used to evaluate how a support seeker and volunteer counselor pair impacted the users; however, retention is an unclear metric in the OMHC context as it can indicate either failure or success of the community. Third, our matching analysis lacks one key type of demographic information: race. As we mentioned in our Related Work section, race, ethnicity, and cultural matching are important factors that clients consider when finding a mental health service provider. However, our data set lacked racial data for a huge majority of users, and we did not conduct automatic detection of racial disclosure within chats. As a result, our findings can only comment on matching from a demographic standpoint on gender and age. Finally, we note our previous mention that optimizing directly for rating, blocking, or other outcome metrics may be an effective and intuitive protocol method for online platforms, but we were not able to experiment with these protocols given our prediction model and evaluation methodology.

### Conclusions

In this paper, we used agent-based modeling in the online mental health context to reveal trade-offs of algorithmically matching peers. Evaluating data from the research site, we provided a simulation model to compare current matching mechanisms and various algorithmic matching policies and observed their differing effects on outcome metrics, including waiting time and chat experiences of support seekers. Our results indicated that algorithmic matching policies based on the applicant-proposing deferred acceptance algorithm can lead to better chat experiences for OMHC support seekers while still matching them for chats quickly. Our simulation can aid designers of OMHCs and other online communities with a need for matching through uncovering the tensions between goals of matching as well as its impact on different communities.

## Appendix

Multimedia Appendix 1 Pseudocode for applicant-proposing deferred acceptance algorithm.

## Abbreviations

HIPAA: Health Insurance Portability and Accountability Act
LGBTQ+: lesbian, gay, bisexual, transgender, and queer
OMHC: online mental health community

## Data Availability

The data sets generated during and analyzed during this study are not publicly available due to data privacy and collaboration agreements; our data set was obtained through collaboration with the anonymous online mental health community. However, we have released our simulation code as open source [51] so that research site stakeholders, other online mental health community designers, and even students can use our simulation sandbox to learn and understand the trade-offs in designing real-world matching algorithms.
