# Supplementary material for: Exploring Trade-Offs for Online Mental Health Matching: Agent-Based Modeling Study
Source: JMIR Form Res. 2024 Oct 1;8:e58241. doi: 10.2196/58241 (PMC11480686; doi:10.2196/58241)
Supplement: Multimedia Appendix 1 [file formative_v8i1e58241_app1.docx]

## Appendix

**Pseudocode for Applicant-Proposing Deferred Acceptance Algorithm**

Parameter S {support-seekers available to be matched in the current simulation period}

Parameter V {volunteer counselors available to be matched in the current simulation period}

function stableMatching {

Initialize all s in S and v in V to be available

while there exists available s who still has a volunteer v to apply to {

v = first volunteer counselor on support seeker s' list to whom v has not yet applied

if v is available

(s, v) become matched

else some pair (s', v) already exists

if v prefers s to s'

s' becomes available

(s, v) become matched

else

(s', v) remain matched

}

}

**Additional outcome measures for protocols by demographic**

|  | **Matching success rate** | **Avg. waiting time (matched)** | **Avg. waiting time (not matched)** |
| --- | --- | --- | --- |
| **Replication of research site** |  |  |  |
| adult | 78.97% | 3.19 | 3.69 |
| under-age | 78.65% | 3.18 | 3.68 |
| non-gender-minority | 78.92% | 3.18 | 3.69 |
| gender-minority | 78.76% | 3.26 | 3.74 |
|  |  |  |  |
| **first come first serve** |  |  |  |
| adult | 81.92%** | 3.68** | 2.73** |
| under-age | 82.02%** | 3.70** | 2.72** |
| non-gender-minority | 81.96%** | 3.69** | 2.73** |
| gender-minority | 81.60%** | 3.68** | 2.73** |
| **last come first serve** |  |  |  |
| adult | 74.85%** | 2.61** | 4.71** |
| under-age | 74.64%** | 2.62** | 4.60** |
| non-gender-minority | 74.81%** | 2.62** | 4.68** |
| gender-minority | 74.75%** | 2.58** | 4.87** |
| **similarity** |  |  |  |
| adult | 79.27% | 3.35** | 3.52** |
| under-age | 79.78%* | 3.28** | 3.55* |
| non-gender-minority | 80.09%** | 3.31** | 3.49** |
| gender-minority | 67.81%** | 3.86** | 3.83 |
| **- age-based** |  |  |  |
| adult | 81.26%** | 3.35** | 3.33** |
| under-age | 74.08%** | 3.65** | 3.65** |
| non-gender-minority | 79.98% | 3.40** | 3.40** |
| gender-minority | 79.86% | 3.43** | 3.51* |
| **- gender-based** |  |  |  |
| adult | 81.80%** | 3.61** | 2.87** |
| under-age | 81.88%** | 3.61** | 2.86** |
| non-gender-minority | 82.31% | 3.61 | 2.81 |
| gender-minority | 74.07% | 3.64 | 3.41 |
| **- topic** |  |  |  |
| adult | 80.66% | 3.45 | 3.30 |
| under-age | 80.90%** | 3.49 | 3.24 |
| non-gender-minority | 80.67% | 3.45 | 3.29 |
| gender-minority | 81.24%* | 3.49 | 3.29 |
| **filter** |  |  |  |
| adult | 56.07% | 3.54 | 3.90 |
| under-age | 85.53%** | 2.23 | 3.28 |
| non-gender-minority | 59.85% | 3.32 | 3.85 |
| gender-minority | 84.48% | 2.20 | 4.02 |

*P < .05

**P<.001
